# Identification and Characterization of a Thermostable GH36 α-Galactosidase from *Anoxybacillus* *vitaminiphilus* WMF1 and Its Application in Synthesizing Isofloridoside by Reverse Hydrolysis

**DOI:** 10.3390/ijms221910778

**Published:** 2021-10-05

**Authors:** Jialing Wang, Xuefei Cao, Weihao Chen, Jiaxing Xu, Bin Wu

**Affiliations:** 1 College of Biotechnology and Pharmaceutical Engineering, Nanjing Tech University, 30 Puzhunan Road, Nanjing 211816, China; wjl_9879@163.com (J.W.); 201961118003@njtech.edu.cn (X.C.); cwh136475399@163.com (W.C.); 2 School of Pharmaceutical Sciences, Nanjing Tech University, 30 Puzhunan Road, Nanjing 211816, China; 3 College of Chemistry and Chemical Engineering, Huaiyin Normal University, 111 Jiangxi Road, Huai’an 223300, China

**Keywords:** α-Galactosidase, *Anoxybacillus vitaminiphilus*, thermal-stability, reverse hydrolysis, isofloridoside

## Abstract

An α-galactosidase-producing strain named *Anoxybacillus vitaminiphilus* WMF1, which catalyzed the reverse hydrolysis of d-galactose and glycerol to produce isofloridoside, was isolated from soil. The α-galactosidase (galV) gene was cloned and expressed in *Escherichia coli*. The galV was classified into the GH36 family with a molecular mass of 80 kDa. The optimum pH and temperature of galV was pH 7.5 and 60 °C, respectively, and it was highly stable at alkaline pH (6.0–9.0) and temperature below 65 °C. The specificity for p-nitrophenyl α-d-galactopyranoside was 70 U/mg, much higher than that for raffinose and stachyose. Among the metals and reagents tested, galV showed tolerance in the presence of various organic solvents. The kinetic parameters of the enzyme towards p-nitrophenyl α-d-galactopyranoside were obtained as *K*_m_ (0.12 mM), *V*_max_ (1.10 × 10^−3^ mM s^−1^), and *K*_cat_*/K*_m_ (763.92 mM^−1^ s^−1^). During the reaction of reverse hydrolysis, the enzyme exhibited high specificity towards the glycosyl donor galactose and acceptors glycerol, ethanol and ethylene glycol. Finally, the isofloridoside was synthesized using galactose as the donor and glycerol as the acceptor with a 26.6% conversion rate of galactose. This study indicated that galV might provide a potential enzyme source in producing isofloridoside because of its high thermal stability and activity.

## 1. Introduction

α-Galactosidases (α-d-galactoside galactohydrolases; EC 3.2.1.22) are exoglycosidases that catalyze the hydrolysis of the terminal non-reducing α-galactosyl residue of various substrates [1]. α-Galactosidases have been classified into six glycoside hydrolase (GH) families namely GH4, GH27, GH36, GH57, GH97, and GH110 based on structure and sequence similarity, but most of them belong to the GH27 or GH36 families, which share common evolutionary origins and reaction mechanism [2]. A majority of GH36 α-galactosidases are reported from bacterial sources with high molecular mass and multimeric nature [3]. α-Galactosidases are known to be potentially useful in diverse applications. In the pharmaceutical industry, they have been shown to be effective against Fabry disease [4]. Additionally, α-galactosidases, which act as hydrolases in nature, can be used in the food industry, such as the hydrolysis of galactosyl residues from raffinose to improve the crystallization of sucrose [5]. Studies conducted over the years have shown that α-galactosidases can mediate transglycosylation to produce a series of important compounds [6].

α-d-Galactosyl-glycerol is the main photosynthetic assimilation product in red algae, which plays an important role in regulating osmotic pressure [7]. Two structural types of α-d-galactosyl-glycerol exist, namely 2-*O*-α-d-galactopyranosyl-glycerol (floridoside) and 1-*O*-α-d-galactopyranosyl-glycerol (isofloridoside), which are widely used in cosmetics, healthcare, food and medicine because of their chemical antioxidant, anti-inflammatory, immune-regulatory and free radical-scavenging effects [8]. High concentrations of isofloridoside have been found in red algae (Hydra and Porphyra), but using alcohol for extracting isofloridoside was complex with low efficiency. Moreover, the chemical method to synthesize galactosyl glycerol requires complicated steps and highly toxic bromo glycosides [9].

Enzymatic synthesis can overcome these shortcomings. Two methods were developed for the enzymatic synthesis of galactosyl glycerol, namely kinetically controlled transglycosylation and thermodynamically controlled reverse hydrolysis [10]. The transglycosylation reaction is fast, but it needs expensive galactosyl donors such as melibiose or pNP-α-d-galactopyranoside and the products are difficult to separate and purify. In addition, the transglycosylation reaction requires a high concentration of the substrate to make the reaction proceed in the direction of synthesis, and the presence of water will cause the hydrolysis of the product, leading to low synthetic efficiency. For example, α-galactosidase from *Penicillium oxalicum* SO catalyzed the transglycosylation of melibiose and glycerol with the reaction time of 80 h, which produced not only galactosylglycerol, but also tetrasaccharides from the self-transglycosylation of melibiose [11]. In previous studies, the cheap ingredient guar gum was used as the donor for synthesizing galactosyl glycerol. However, the enzyme-catalyzed transglycosylation was complex, and contained not only α-galactosidase, but also β-mannosidase and β-mannanase, while α-galactosidase alone could not catalyze the synthesis of galactosyl glycerol from guar gum by transglycosylation [12]. In contrast, the reverse hydrolysis needed low-cost substrates such as glycerol and galactose, and the product was single, which is more conducive to the industrial production of galactosyl glycerol in the future [10]. So far, there has been only one report which used whole-cell biocatalysts harboring α-galactosidase to catalyze the reverse hydrolysis of galactose and glycerol to synthesize isofloridoside [13]. However, since the reverse hydrolysis reaction needs a long reaction time and the efficiency is low, it is necessary to find more enzymes with high synthetic ability.

Since the reverse hydrolysis reaction is controlled by thermodynamics, high temperature is more conducive to the reaction. In addition, increasing the temperature can accelerate the reaction rate, making the reaction reach equilibrium earlier because the reaction of reverse hydrolysis is relatively slow [14]. Moreover, high temperature will increase the solubility of substrates and the initial efficiency of enzymes, thereby increasing the yield [15]. Thus, α-galactosidases characterized by thermal stability are considered to have good potential in synthesizing galactosyl glycerol. To date, α-galactosidases with thermal stability from thermophilic fungi and prokaryotic sources are few, especially those able to catalyze the synthetic reaction.

**Figure 1:**
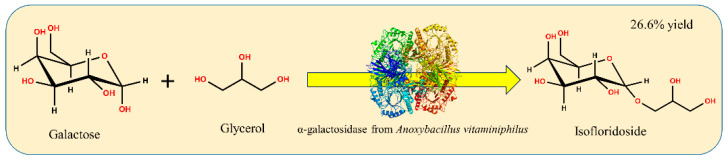
Synthesis of isofloridoside from galactose and glycerol by reverse hydrolysis catalyzed by galV.

Here, the microorganism that could catalyze the synthesis of isofloridoside with reverse hydrolysis was isolated from soil and identified as *Anoxybacillus vitaminiphilus*. An α-galactosidase (galV) gene from *A. vitaminiphilus* WMF1 was cloned, expressed in *Escherichia coli*, and characterized. Furthermore, galV was used to catalyze the reverse hydrolysis of galactose and glycerol to synthesize isofloridoside (Figure 1).

## 2. Results and Discussion

### 2.1. Obtaining α-Galactosidase-Secreting Strains

Six α-galactosidase-producing strains were found by observing the blue single colony on the primary screening plate, and enzyme activity was detected in the supernatant of lysate but not in the supernatant of fermentation. Among them, a strain with the highest activity to catalyze the synthesis of isofloridoside by reverse hydrolysis was selected. The 16s rDNA sequence of the target strain, which was named as *A. vitaminiphilus* WMF1, possessed the highest homology (99.0%) with that of *A. vitaminiphilus* (NCBI GenBank accession no. NR_108379). As far as we know, no report has been published on α-galactosidase from *Anoxybacillus* sp. Therefore, the research on the biochemical characterizations of α-galactosidase from *Anoxybacillus* sp. is of great significance.

### 2.2. Sequence and Structure Analysis

**Figure 2:**
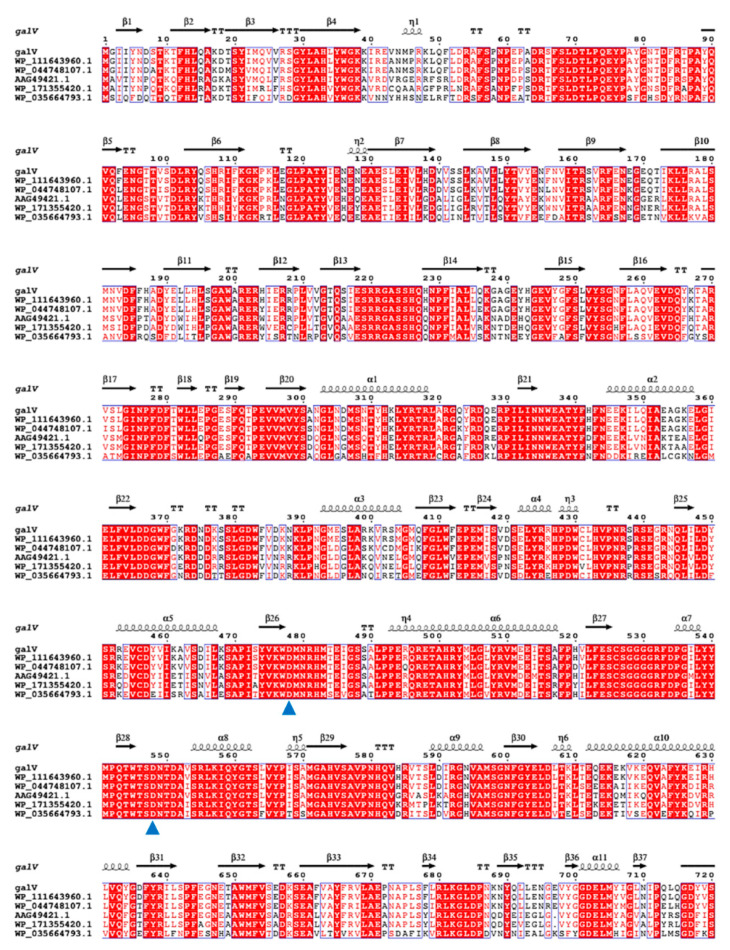
Multiple sequence alignment of galV and its homologs. Proteins in the alignment are GH36 α-galactosidases from *Anoxybacillus vitaminiphilus*, *Bacillus alveayuensis*, *Geobacillus* sp. MR, *Alkalihalobacillus akibai* and *Geobacillus stearothermophilus*. Blue triangles indicate putative nucleophile residues and acid/base catalytic residues.

The genomic DNA sequence of *A. vitaminiphilus* was found in NCBI, and a putative α-galactosidase gene was noted; however, the gene has not been cloned, expressed, and characterized yet. According to this sequence, the primers were designed and then the α-galactosidase gene from *A. vitaminiphilus* WMF1 was cloned. The sequencing analysis showed that galV showed 75.3% identity with the putative α-galactosidase from *A. vitaminiphilus* (WP_111643960.1). Additionally, galV shared the identity of 91.4% with the uncharacterized α-galactosidase from *Bacillus alveayuensis* (WP_044748107.1), followed by the α-galactosidase from *Geobacillus* sp. MR (73.4%, WP_171355420.1) and *Alkalihalobacillus akibai* (67.0%, WP_035664793.1). The α-galactosidase from *G. stearothermophilus* (AAG49421.1), which has been experimentally characterized, shared 75.3% identity with galV and was used as the template for modeling (Figure 2). The theoretical molecular mass of galV was calculated to be 83.8 kDa. No signal peptide was found in galV, which was consistent with the aforementioned result that the α-galactosidase was an intracellular enzyme. A catalytic domain belonging to GH36 α-galactosidase was observed in the sequence (from Glu328 to Glu627), indicating that galV should be a GH36 family α-galactosidase. Moreover, galV was found to contain the consensus motif LFVL/MDDGWFG of GH36 family α-galactosidases [16]. Residues D478 and D548 are the putative nucleophile and catalytic acid/base in the motif KWD and SDXXDXXXR of galV, respectively [16].

**Figure 3:**
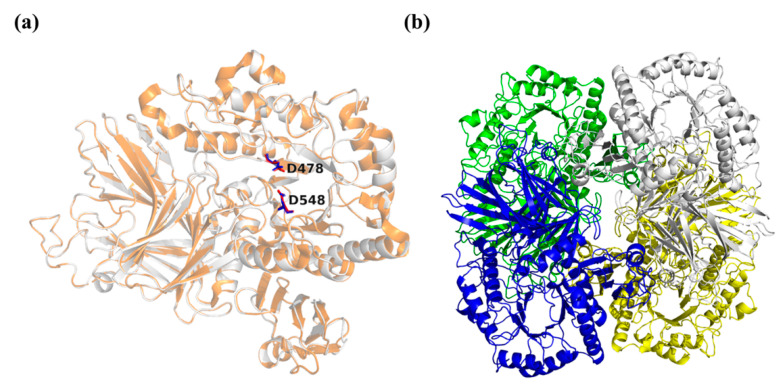
Overall structure of galV. (**a**) Comparison of the quaternary structure of galV (white) and the template (yellow). Sites D478 and D548 are red sticks in galV and blue sticks in the template. (**b**) A schematic representation of the tetrameric α-galactosidase.

The putative structure of galV showed the typical GH36 organization, which comprised three parts: *N*-terminal domain, catalytic domain with a conserved (β/α)_8_-barrel topology and similar active sites, and C-terminal domain [17]. The *N*-terminal domain (residues 1–327), which was connected to the catalytic domain, consisted of a β-super sandwich and terminated in a long α-helix. The catalytic domain (residues 328-627) showed a (β/α)_8_-barrel fold containing the putative nucleophile and proton donor, Asp478 and Asp548 (Figure 3). The least conservative of the three domains, the C-terminal domain (residues 628-727), showed a β-sandwich structure, which contained an α-helix and eight β-folds. Furthermore, galV was presumed to have a symmetrical tetramer structure because the template α-galactosidase was tetrameric, which was observed in a previous study [17].

### 2.3. Expression and Purification of galV

**Figure 4:**
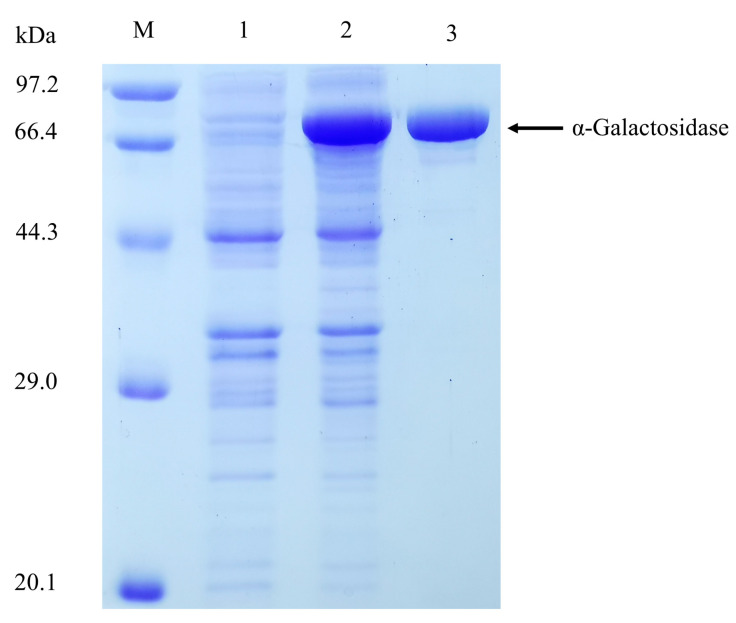
SDS-PAGE analysis of α-galactosidase expressed in *E. coli* BL21 (DE3). Lanes: M, protein molecular weight marker; 1, pET-28a; 2, crude pET-28a-α-galactosidase; 3, purified pET-28a-α-galactosidase.

Recombinant α-galactosidase was abundantly expressed in *E. coli*. The recombinant protein was approximately 80 kDa on a 12% SDS-PAGE gel, which was in agreement with the calculated molecular mass (83.8 kDa) of galV (Figure 4) and in the range of the molecular weight (70–100 kDa) of most GH36 α-galactosidases [18]. The native molecular mass of the enzyme was 320 kDa as determined by gel filtration, suggesting a homotetramer structure, which was consistent with the previously speculated structure, and the same result was also found in the α-galactosidase from *Paecilomyces thermophila* [19].

### 2.4. Biochemical Characterization of galV

**Figure 5:**
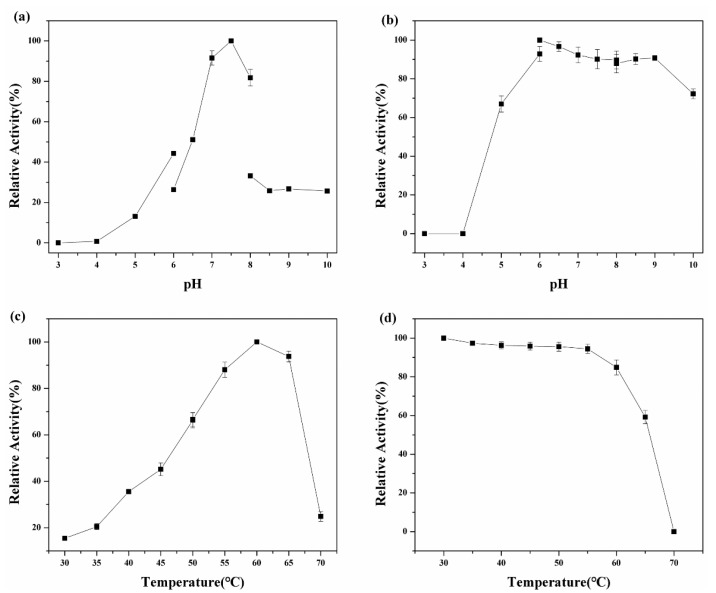
Effects of pH and temperature on enzyme activity and stability. (**a**) Optimal pH, (**b**) pH stability, (**c**) optimal temperature and (**d**) thermal stability of α-galactosidase.

The optimal pH of galV was found to be approximately 7.5, which was consistent with the previous finding that GH36 enzymes functioned optimally at neutral or alkaline pH [20]. Figure 5 shows that galV manifested the maximum activity at 60 °C, which was in agreement with the α-galactosidase from *Paceilomyces thermophila* [19] and higher than that reported for α-galactosidase from *Bifidobacterium breve* (37 °C) [21] and *Aspergillus oryzae* YZ1 (50 °C) [22]. Under the optimal conditions, galV showed a specificity of 70 U/mg against pNPG, which was higher than that of α-galactosidase from *Carnobacterium piscicola* (2.3 U/mg) [23] and *Lactobacillus fermenti* (2.19 U/mg) [24], but lower than that produced by *Aspergillus oryzae* YZ1 (76.9 U/mg) [22] and *Penicillium janczewskii zalesk* (667 U/mg) [25].

The enzyme was stable over a slightly alkaline pH range between 6.0 and 9.0, which was consistent with the α-galactosidase from *Bacillus stearothermophilus* NCIM 5146 [26]. Contrary to our results, the α-galactosidase from *Penicillium* sp. F63 CGMCC 1669 [16] and *Penicillium janczewskii zaleski* [27] had optimum activity in the acidic pH range. The neutral or weak alkaline pH form of α-galactosidase is suitable for the hydrolysis of soy milk, since an acidic pH leads to the deposition of soy protein and gives milk its sour taste [26]. The thermostability of galV was also measured. About 78% of its original activity was retained after incubation at 60 °C for 2 h, which was consistent with the α-galactosidase from *thermophilic microorganisms*, such as the α-galactosidase from *Rhizomucor miehei* [28] and *Dictyoglomus thermophilum* sp [29]. Moreover, galV was more stable than most GH36 α-galactosidases, such as the α-galactosidase from *Bacillus megaterium* [18], *Yersinia pestisbiovar Microtus str*. 91,001 [30], *Aspergillus oryzae* YZ1 [22] and *Paceilomyces thermophila* [19]. Thus, the enzyme showed activity and stability over a broad range of temperature, which made it a potential candidate in various industrial processes.

Table 1 presents the effects of metal ions and reagents on galV. The enzyme activity drastically decreased to 2.37%, 4.86%, and 3.93% of the original activity in the presence of Fe^2+^, Ni^2+^, and Fe^3+^, respectively, while Ca^2+^, Mn^2+^, and Zn^2+^ considerably inhibited the activity. The drastic mitigation of galV activity was seen in the presence of Cu^2+^ (0.16% residual activity), which was also reported for the α-galactosidase from *Aspergillus terrus_GR_* [31]. Na^+^, K^+^, Li^+^, and Mg^2+^ did not affect the enzymatic action, which was similar to that observed in *Humicola* sp [32] and *Alicyclobacillus* sp. A4 [33]. Reagents such as CTAB, SDS, and acetonitrile had a strong inhibitory effect on the enzyme activity. Most proteins lose the tertiary and quaternary structures under the action of SDS due to the strong denaturation of SDS [34]. Unlike the α-galactosidase from *Bacillus megaterium*, the organic solvents DMSO and methanol had no significant effect on the enzyme activity [18]. The tolerance of galV to alcohol may make it easier to construct a solvent-free system. In this case, a high concentration of acceptor such as alcohol is beneficial to the reverse hydrolysis reaction, resulting in a high yield.

The kinetic parameter values of the galV were obtained using the Lineweaver–Burk plot with certain concentrations of pNPG. The *K*_m_, *V*_max_, and *K*_cat_*/K*_m_ for pNPG were 0.12 mM, 1.10 × 10^−3^ mM s^−1^, and 763.92 mM^−1^ s^−1^, respectively. The kinetic parameters of α-galactosidases have been studied extensively. Accordingly, the catalytic efficiency (*K*_cat_*/K*_m_) of galV toward pNPG was forty-fold that of the α-galactosidase from *Rhizomucor miehei* [28]. Furthermore, the α-galactosidase from *Bacillus megaterium* possessed *K*_m_ and *K*_cat_*/K*_m_ values of 0.42 mM and 610 mM^−1^ s^−1^, respectively [18], and the α-galactosidase from *Sphingomonas* sp. had the *K*_m_ of 2.2 mM and *K*_cat_*/K*_m_ of 233 mM^−1^ s^−1^ [35]. In addition, the α-galactosidase from *Irpex lacteus* owned the *K*_m_ of 1.2 mM and *K*_cat_*/K*_m_ of 1900 mM^−1^ s^−1^ [36]. Compared with other α-galactosidases, galV was moderate in its activity to catalyze the hydrolysis of pNPG.

### 2.5. Substrate Specificity in the Hydrolysis Reaction

The substrate specificity of galV was tested on the artificial substrates: pNPαGal and pNPβGal, and the natural substrates: melibiose, raffinose, stachyose, lactose, D(+)-cellobiose and guar gum. Further, galV showed no activity on pNPβGal, lactose, and D(+)-cellobiose, indicating that aglV was highly specific for α-1,6-bound galactose. Moreover, galV showed remarkably higher activity towards pNPαGal than toward the natural substrate (Table 2). The result was in agreement with previous findings (Table 3). Most α-galactosidases exhibited higher activity with synthetic substrates (pNPG) than with natural substrates (melibiose, raffinose, and stachyose) [37], which might be because of the simple structure of pNPαGal [38]. For the galacto-oligosaccharide substrates investigated, galV exhibited negligible activities on raffinose (2.75%) and stachyose (1.50%) compared with pNPG (100%), but did not show any activity on melibiose. It was different from many α-galactosidases, which showed activity on melibiose to different degrees, such as the α-galactosidase from *Lichtheimia ramosa* [39] and *Penicillium* sp. F63 CGMCC 1669 [16]. Like most GH36 α-galactosidases, the enzyme did not act on polymeric galactomannan guar gum. In previous studies, α-galactosidases belonging to the GH36 family were specific for small oligosaccharides but inactive on galactomannans, while GH27 α-galactosidases could hydrolyze galactomannans [40].

**Figure 6:**
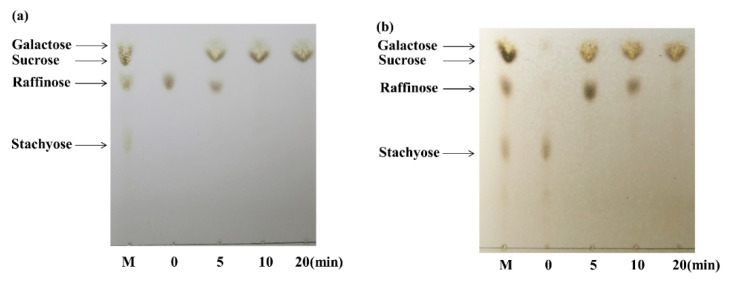
TLC analysis of the hydrolysis products of raffinose (**a**) and stachyose (**b**) by galV. Lane M, a mixture of galactose, sucrose, raffinose, and stachyose.

As shown in Figure 6, the degradation of raffinose and stachyose by galV was performed and analyzed by TLC. Most of the raffinose was rapidly degraded into sucrose and galactose in 5 min, and the residue was completely hydrolyzed in 10 min (Figure 6a). For the hydrolysis of stachyose, the degradation of the tetrasaccharide stachyose produces the intermediate trisaccharide raffinose in the initial hydrolysis process, which indicates that galV is an exoglycosidase [42]. The formed raffinose was completely converted to galactose and sucrose as the final product in 20 min (Figure 6b). The difference in efficiency of hydrolysis of the two oligosaccharides catalyzed by galV was in agreement with the result that galV showed higher substrate specificity for raffinose than stachyose. The complete hydrolysis of raffinose was faster than that of stachyose, probably because there is one more α-1, 6-galactose bond in stachyose than in raffinose [36].

### 2.6. Reverse Hydrolysis of galV

The capability of galV to synthesize glycosides by reverse hydrolysis was investigated, using sugars as a donor and alcohols and sugar alcohols as an acceptor (Table 4). The result indicated that d-galactose and glycerol were the best substrates for reverse hydrolysis catalyzed by galV. No synthetic product was observed but ethanol and ethylene glycol were used as acceptors, with similar relative galactose conversion rates (about 85.2% and 88.6% respectively). Many studies showed that glycerol was a good acceptor. For instance, α-galactosidase from *Penicillium oxalicum* SO exhibited high acceptor specificity towards glycerol [11]. In addition, a previous study indicated that mono-alcohols were a poor acceptor compared with ethylene glycol and glycerol [12]. However, in our study, ethanol was also a good acceptor. Therefore, this enzyme has great potential for application in synthesizing alkyl glycosides.

### 2.7. Synthesis of Isofloridoside

**Figure 7:**
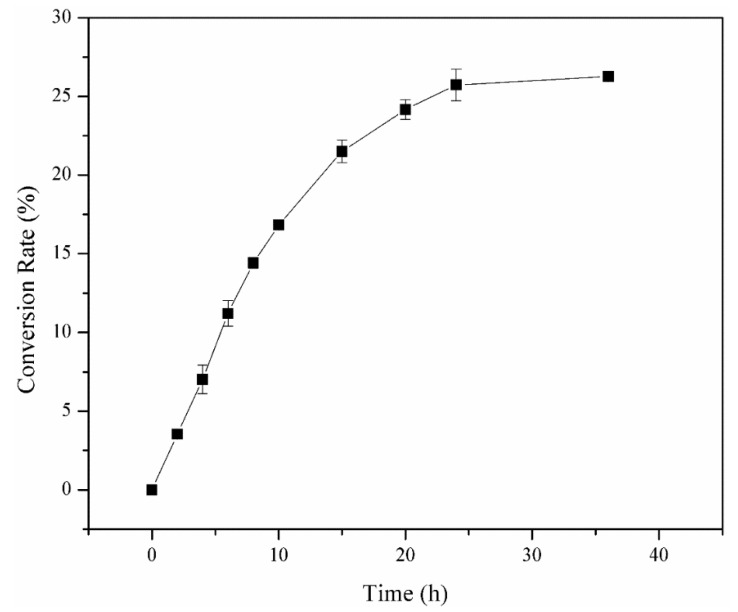
Time-course during the synthesis of isofloridoside with the α-galactosidase (data from HPLC analysis).

**Figure 8:**
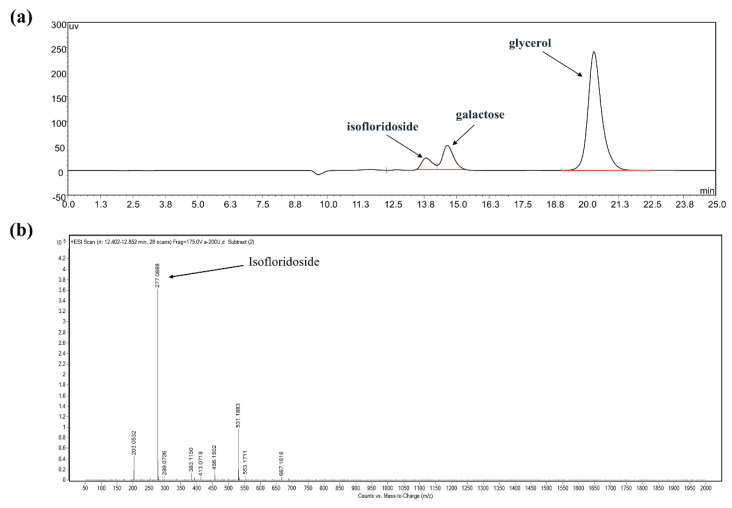
(**a**) HPLC analysis of the reaction mixture from reverse hydrolysis catalyzed by galV. (**b**) LC−MS spectra of the reaction mixture from reverse hydrolysis catalyzed by galV.

Figure 7 shows the time-course for synthesizing isofloridoside using the low-cost ingredients d-galactose and glycerol. The content of d-galactose and glycerol decreased with the extension of reaction time, resulting in a gradual increase in the content of isofloridoside in the time progression of synthesis. There was no significant increase in the content of isofloridoside after reaction for 24 h. The final conversion rate of galactose was 26.6% without the optimization of reaction conditions (Figure 8a). The structure of isofloridoside was identified by LC−MS (Figure 8b). Mass spectra showed a peak with [M+Na]^+^ molecular ions of 277.0, which confirmed that the product was isofloridoside (m/z 254). In addition, galactosyl glycerol was synthesized by transglycosylation, using activated sugar melibiose or pNPG as the substrate [43]. However, the use of these expensive substrates is not practical in producing galactosyl glycerol. On the contrary, the reverse hydrolysis reaction does not require activated sugar, the ingredients needed are cost effective, and the product is single, which is more suitable for industrial production. Wang used the α-galactosidase from *Alicyclobacillus hesperidum* to catalyze the synthesis of isofloridoside by reverse hydrolysis, the galactose conversion was 23% after optimizing pH, temperature, and galactose and glycerol concentration [13]. In the future, effective methods should be used to improve the content of isofloridoside, making it more suitable for expanding production, such as protein engineering on the enzyme and optimization of the reaction parameters.

## 3. Experimental Procedures

### 3.1. Materials

*E. coli* DH5α and pMD 19-T vector for gene cloning, and *E. coli* BL21 (DE3) and pET-28a (+) for gene expression of α-galactosidase were preserved in our laboratory. Restriction endonuclease, pfu DNA polymerase and T4 DNA ligase were purchased from TaKaRa (Tokyo, Japan). Genomic DNA and plasmid extraction kits were obtained from Bioteke (Beijing, China). The substrates pNP-α-d-galactopyranoside (pNPG), pNP-β-d-galactopyranoside, melibiose, raffinose, stachyose, lactose, D(+)-cellobiose and guar gum were purchased from Sigma Chemical Company (MO, USA). The alcohols (methanol, ethanol, ethylene glycol (1,2-ethanediol)) and sugar alcohols (xylitol, inositol, d-sorbitol and mannitol) were purchased from Sinopharm Chemical Reagent Co., Ltd. (Shanghai, China). All other chemicals used were of analytical grade unless otherwise stated.

### 3.2. Microorganism Isolation and Identification

Soil samples from the Laoshan Forest Park (Nanjing, China) were collected. For obtaining the enriched culture, the soil samples were placed in sterile water and stirred at 37 °C. After 30-min standing, the supernatant was inoculated into the liquid medium containing 1% (NH_4_)_2_SO_4_, 0.5% NaOAc, 0.2% citrate diamine, 0.2% KH_2_PO_4_, 2% raffinose and 0.058% MgSO_4_·7H_2_O. Subsequently, the microorganisms were gradually diluted with sterile water and screened using a primary screening medium (1% tryptone, 1% beef extract, 0.5% NaOAc, 0.2% citrate diamine, 0.2% KH_2_PO_4_, 0.5% yeast extract, 2% raffinose, 0.058% MgSO_4_·7H_2_O, 2% agar and 6.25 × 10^−3^% X-α-Gal). The α-galactosidase-producing colonies were screened through blue and white spots and cultured on the fermentation medium (1% tryptone, 1% beef extract, 0.5% NaOAc, 0.2% citrate diamine, 0.2% KH_2_PO_4_, 0.5% yeast extract, 2% raffinose and 0.058% MgSO_4_·7H_2_O). The hydrolysis activity of the supernatant of fermentation and the sediment strains lysed by ultrasonication on ice (work time: 15 min, work/interval time: 3 s/5 s and ultrasonic output power: 200 W) was assayed using pNPG as substrate. For isofloridoside synthesis, screw-capped glass vials were used for carrying out reverse hydrolysis between glycerol and galactose at 35 °C for 24 h. The strain with the highest activity for synthesizing isofloridoside was identified based on the analysis of 16S rDNA sequence Basic Local Alignment Search Tool (BLAST) in the GenBank Data Library using primers 27F (5′-AGAGTTTGATCCTGGCTCAG-3′) and 1492R (5′-GGTTACCTTGTTACGACTT-3).

### 3.3. Cloning, Sequence and Structure Analysis of α-Galactosidase Gene

The galV-encoding gene was amplified using the forward primer (5′-CATGCCATGGTTAGCACGCCTTCAGCCTCC-3′ with restriction *Ncol*Ⅰ site) and reverse primer (5′-CCGCTCGAGATGGGGATTATATATAATGA-3′ with restriction *Xho*Ⅰ site), and ligated to the pMD 19-T vector, transformed into *E. coli* DH5α and sequenced by GENEWIZ, Inc (Suzhou, China). The plasmid was digested with *Ncol*Ⅰ and *Xho*Ⅰ and ligated to the pET-28a (+), which was used as an expression backbone and transformed into *E. coli* BL21 (DE3).

The presence of signal peptides was detected using SignalP 5.0 (http://www.cbs.dtu.dk/services/SignalP/, accessed on 13 March 2021). Protein homology searches were conducted by BLASTX (https://blast.ncbi.nlm.nih.gov/Blast.cgi, accessed on 13 March 2021) and ClustalW (https://www.genome.jp/tools-bin/clustalw, accessed on 13 March 2021) was used for sequence alignments. Conserved domains were analyzed at National Center for Biotechnology Information (NCBI) CD-Search (https://www.ncbi.nlm.nih.gov/Structure/cdd/wrpsb.cgi, accessed on 16 April 2021). The classification of enzyme into a GH family was determined by InterPro (http://www.ebi.ac.uk/interpro/, accessed on 16 April 2021). The theoretical molecular mass of the recombinant protein was predicted using the ExPASy ProtParam tool.

The three-dimensional structure of α-galactosidase from *A. vitaminiphilus* AWM1 was determined with a Swiss Model server using the α-galactosidase from *Geobacillus stearothermophilus* (PDB-ID: 4fnq) as the template and optimized based on the energy minimization.

### 3.4. Expression and Purification of Enzymes

The inoculum was prepared by transferring loopfuls of fresh strains cultured on a Luria broth (LB) agar plate into an LB medium containing kanamycin, followed by incubation at 37 °C for 12 h. The inoculation amount of 2% (*v/v*) was transferred to a fresh LB medium containing kanamycin for 90 min at 37 °C and induced with Isopropyl β-d-Thiogalactoside (IPTG) at a final concentration of 0.1 mM at 20 °C for 20 h. The sediment strain of the culture broth was resuspended in 50 mM Na_2_HPO_4_–NaH_2_PO_4_ (pH 7.5) after centrifugation (12000 rpm, 4 °C, 20 min) and lysed by ultrasonication on ice (work time: 10 min, work/interval time: 3 s/5 s and ultrasonic output power: 200 W). The supernatant of total lysate, which was directly used as crude α-galactosidase for purification, was obtained by centrifugation.

The crude α-galactosidase was purified by nickel affinity chromatography following the manufacturer’s protocols. Purified α-galactosidases were analyzed by 12% sodium dodecyl sulfate-polyacrylamide gel electrophoresis (SDS-PAGE). The native molecular mass was estimated using a HiLoad 16/600 Superdex 200 pg gel filtration column (GE Healthcare, USA). The molecular mass standards used in gel filtration included thyroglobulin (669 kDa), ferritin (440 kDa), aldolase (158 kDa), conalbumin (75 kDa) and ovalbumin (44 kDa). The protein concentrations were determined using the Bradford method.

### 3.5. Enzyme Assay

pNPG (10 mM) was incubated with an enzyme sample in 50 mM NaH_2_PO_4_–Na_2_HPO_4_ buffer (pH 7.5) at 35 °C for 10 min (working volume of 250 μL). Then the absorbance of the released p-nitrophenol at 410 nm was determined. One unit of enzyme activity (U) was defined as the amount of the enzyme required to liberate 1 μmol of p-nitrophenol per minute. The enzyme was deactivated by boiling for 5 min.

### 3.6. Biochemical and Kinetic Properties of galV

For optimal pH, the enzyme activity was measured at various pH values ((pH 3–10), using citrate buffer (pH 3–6), phosphate buffer (pH 6–8) and Tris-HCl buffer (pH 8–10)) at 35 °C for 10 min. Under stable pH conditions, the enzyme was pre-incubated at various pH values (pH 3–10) and at 35 °C for 120 min. For optimal temperature, the enzyme activity was measured at various temperatures (30–80 °C) and pH 7.5 for 10 min. Under stable temperature conditions, the enzyme was pre-incubated at various temperatures and pH 7.5 for 120 min.

The effects of different metal ions on galV activity were determined by incubating the enzyme with 100 mM solution of Na^+^, K^+^, Li^+^, Ni^2+^, Ba^2+^, Ca^2+^, Cu^2+^, Fe^2+^, Mg^2+^, Mn^2+^, Zn^2+^ or Fe^3+^ for 1 h at room temperature. The effects of additives on α-galactosidase were determined by incubating the enzyme with 1% solution of SDS, cetyltrimethylammonium bromide (CTAB), Triton X-100, Tween-80, dimethyl sulfoxide (DMSO), methanol and acetonitrile for 1 h at room temperature.

Kinetic parameters were determined by performing enzymatic reactions at 35 °C, with pNPG (0.01–20 mM) in 50 mM NaH_2_PO_4_–Na_2_HPO_4_ buffer (pH 7.5) as the substrate. The products were monitored as described earlier, and the reaction rate was calculated. The catalytic constant (*K*_cat_) and specificity constant (*K*_cat_/*K*_m_) were calculated using *K*_m_ and *V*_max_ determined from the Lineweaver–Burk plot.

### 3.7. Substrate Specificity in a Hydrolysis Reaction

The substrate specificity of galV towards artificial substrates (pNPG and pNP-β-d-galactopyranoside) was measured in the standard assay as described above. For natural substrates, the reaction mixture consisting of 10 mM oligosaccharide or 0.1% guar gum in 50 mM NaH_2_PO_4_–Na_2_HPO_4_ buffer (pH 7.5) was incubated at 35 °C for 30 min (working volume of 2 mL). When raffinose, stachyose and guar gum were used as substrates, the enzyme activity was determined by measuring the reducing sugar using 3,5-dinitrosalicylic acid (DNS) method with galactose as a standard [44]. For melibiose, lactose and D(+)-cellobiose, the released glucose was determined by the glucose oxidase-peroxidase method with a commercial kit (Biosino, Beijing, China). One unit of the enzyme activity was defined as the amount of enzyme required to produce 1 μmol of reducing sugar or glucose per minute.

The hydrolysates of raffinose and stachyose were analyzed. A mixture of purified galV (4.7 unites/mL) and 10 mM raffinose or 10 mM stachyose in 50 mM NaH_2_PO_4_–Na_2_HPO_4_ buffer (pH 7.5) was incubated at 35 °C for 30 min. Aliquots of the solution were sampled at different intervals and boiled for 5 min. Then the reaction products were analyzed by thin-layer chromatography (TLC). Hydrolysates were loaded on silica gel G plates (10 cm × 10 cm) and developed twice using n-propanol/acetic acid/water (10:15:1, *v/v/v*). The plate was sprayed with a mixture of methanol:sulphuric acid (4:1), followed by heating at 115 °C for 10 min to detect sugar spots.

### 3.8. Substrate Specificity in a Reverse Hydrolysis Reaction

The synthetic substrate specificity of α-galactosidase was investigated by mixing 0.3 M donor, 3 M acceptor, and 5 units/mL of the enzyme, giving a final volume of 10 mL by adding 50 mM Na_2_HPO_4_–NaH_2_PO_4_ (pH 7.5). The reaction mixture was incubated at 35 °C for 24 h. It was then boiled for 5 min to deactivate the enzyme after incubating for 24 h. When d-galactose was used as the donor, the acceptors were alcohols (methanol, ethanol, ethylene glycol (1,2-ethanediol), glycerol and 1-butanol) and sugar alcohols (xylitol, inositol, D–sorbitol and mannitol). The donors used were sugars (d-galactose, d-(-)-arabinose, d-xylose, d-fructose, L-sorbose, *N*-acetyl-d-glucosamine, and glucose) with glycerol as the acceptor. The products were evaluated by HPLC. All reactions were performed in triplicate. The results were reported as mean ± standard deviation (SD).

### 3.9. Time-Course for Isofloridoside Synthesis

Galactose (0.3 M), glycerol (3 M) and 5 units/mL of the enzyme were mixed, and 50 mM Na_2_HPO_4_–NaH_2_PO_4_ (pH 7.5) buffer was added to make the volume of the reaction solution 10 mL. The reaction was carried out at 55 °C, and 200 rpm for 36 h, and the samples were taken at regular intervals (0, 2, 4, 6, 8, 10, 15, 20, 24, and 36 h). The samples were transferred to boiling water for 5 min to inactivate the enzyme. After filtration, they were analyzed by high-performance liquid chromatography (HPLC) and liquid chromatography–mass spectrometry (LC−MS).

### 3.10. HPLC Analysis

The sugars formed by the enzymatic reaction were analyzed by HPLC under the following conditions: (1) column, 300 × 7.8 mm^2^, i.d. Aminex HPX-87H (Bio-Rad Ltd., Hercules, CA, USA); mobile phase, 5 mM sulfuric acid; column temperature, 50 °C; flow rate, 1.0 mL/min; and differential refractive index monitor.

## 4. Conclusions

In this study, we successfully discovered and heterologously expressed a thermostable α-galactosidase of the GH36 family from *A. vitaminiphilus* WMF1. galV showed high activity for reverse hydrolysis with d-galactose as the donor and glycerol, ethanol, and ethylene glycol as acceptors. Finally, isofloridoside was synthesized using the low-cost galactose as the donor and glycerol as the acceptor. The conversion rate of galactose was 26.6% without optimization, which provided a potential enzyme to produce isofloridoside. Furthermore, galV could be considered as a good candidate additive for the food and feed industry due to its high thermal stability and tolerance to organic solvents.

## Figures and Tables

**Table 1:** Effects of metal ions and chemical reagents on the activity of α-galactosidase.

Supplement	Relative Activity (%)	Supplement	Relative Activity (%)
None	100 ± 0.03	Mn^2+^	55.13 ± 0.21
K^+^	104.45 ± 0.01	Zn^2+^	15.38 ± 0.08
Li^+^	101.07 ± 0.03	Fe^3+^	3.93 ± 0.07
Na^+^	99.86 ± 0.36	CTAB	8.98 ± 0.14
Ni^2+^	4.86 ± 0.05	SDS	4.43 ± 0.02
Ba^2+^	100 ± 0.07	Triton X-100	100 ± 0.09
Ca^2+^	28.47 ± 0.01	Tween-80	100 ± 0.07
Cu^2+^	0.16 ± 0.00	DMSO	100 ± 0.07
Fe^2+^	2.37 ± 0.08	Methanol	100 ± 0.09
Mg^2+^	99.64 ± 0.06	Acetonitrile	3.43 ± 0.00

The activity in the absence of the supplement was considered as 100%. Values are the mean ± SD of three independent experiments.

**Table 2:** Substrate specificity of the recombinant α-galactosidase.

Substrate	Relative Activity (%)
pNP-α-d-galactopyranoside	100 ± 0.08
pNP-β-d-galactopyranoside	<0.001
Melibiose	<0.001
Raffinose	2.75 ± 0.01
Stachyose	1.50 ± 0.00
Lactose	<0.001
D(+)-cellobiose	<0.001
Guar gum	<0.001

Relative activity was calculated in relation to pNPG activity, which was considered as 100%. Values are the mean ± SD of three independent experiments.

**Table 3:** Thermal and pH stability and substrate specificity of GH36 family α-galactosidases.

Organism	pH Stability	Thermostability	Substrate Specificity	Ref
*Anoxybacillus vitaminiphilus* WMF1	>80%, 6.0–9.0	78%, 60 °C, 2 h	pNPG > raffinose > stachyose > melibiose	This study
*Rhizomucor miehei*	>80%, 4.5–10	70%, 60 °C, 30 min	pNPG > stachyose > raffinose > melibiose	[28]
*Bacillus megaterium*	>70%, 6.0–7.4	80%, 45 °C, 2 h	pNPG > melibiose > raffinose > stachyose	[18]
*Yersinia pestisbiovar Microtus* str. 91001	>60%, 6.5–7.5	63%, 50 °C, 30 min	NR	[30]
*Lichtheimia ramosa*	>65%, 3.0–9.0	90%, 60 °C, 10 min	pNPG > melibiose > raffinose > stachyose	[39]
*Penicillium sp.* F63 CGMCC 1669	5.5–6.5	stable below 40 °C	melibiose > raffinose > stachyose	[16]
*Bacillus stearothermophilus* NCIM 5146	>60%, 6.0–9.0	80%, 65 °C, 2 h	pNPG > melibiose > raffinose > stachyose	[26]
*Bifidobacterium longum* JCM7052	NR	NR	pNPG > raffinose > melibiose > stachyose	[41]
*Dictyoglomus thermophilum* sp	>83%, 7.0–10.0	50%, 60 °C, 12 h	pNPG, melibiose, raffinose, stachyose	[29]
*Meiothermus ruber*	3.0–10.0	50%, 60 °C, 12 h	pNPG, melibiose, raffinose, stachyose	[29]
*Penicillium janczewskii zaleski*	>50%, 4.0–6.8	60%, 35 °C, 2 h	pNPG > melibiose > raffinose > stachyose	[27]
*Aspergillus oryzae* YZ1	>90%, 3.0–8.0	60%, 45 °C, 40 min	pNPG > stachyose > raffinose	[22]
*Paceilomyces thermophila*	>90%, 4.0–11.5	90%, 50 °C, 30 min	pNPG > stachyose > melibiose > raffinose	[19]

NR: not reported.

**Table 4:** Acceptor specificity of α-galactosidase.

Acceptor	Relative Activity (%)
Glycerol	100 ± 0.27
Methanol	<0.001
Ethanol	85.2 ± 0.48
Ethylene glycol	88.6 ± 0.70
1-Butanol	<0.001
Xylitol	<0.001
Inositol	<0.001
D-sorbitol	<0.001
Mannitol	<0.001

Relative activity was calculated in relation to the conversion rate of galactose using glycerol as the acceptor, which was considered as 100%. Values are the mean ± SD of three independent experiments.

## Data Availability

All data included in this study are available from the corresponding author by request.
